# Cell-type-specific ablation of siRNAs by Arabidopsis RTL1 reveals a role of phloem companion cells in systemic post-transcriptional gene silencing

**DOI:** 10.1093/nar/gkag388

**Published:** 2026-04-23

**Authors:** Martin Lacroix, Hayat Sehki, Agnès Yu, Ivan Le Masson, Violette Martinelli, Hervé Vaucheret

**Affiliations:** Universite Paris-Saclay, INRAE, AgroParisTech, Institut Jean-Pierre Bourgin for Plant Sciences, 78000 Versailles, France; Univ. Paris-Sud, Université Paris-Saclay, 91405 Orsay, France; Universite Paris-Saclay, INRAE, AgroParisTech, Institut Jean-Pierre Bourgin for Plant Sciences, 78000 Versailles, France; Univ. Paris-Sud, Université Paris-Saclay, 91405 Orsay, France; Universite Paris-Saclay, INRAE, AgroParisTech, Institut Jean-Pierre Bourgin for Plant Sciences, 78000 Versailles, France; Universite Paris-Saclay, INRAE, AgroParisTech, Institut Jean-Pierre Bourgin for Plant Sciences, 78000 Versailles, France; Universite Paris-Saclay, INRAE, AgroParisTech, Institut Jean-Pierre Bourgin for Plant Sciences, 78000 Versailles, France; Universite Paris-Saclay, INRAE, AgroParisTech, Institut Jean-Pierre Bourgin for Plant Sciences, 78000 Versailles, France

## Abstract

Small RNAs regulate numerous biological processes, including intercellular communication and inter-kingdom relationships, yet their tissue-specific functions remain poorly understood. Dissecting these roles requires tools that selectively eliminate small RNAs in defined cell types. Virus-encoded proteins such as P19 have been widely used to neutralize small RNAs owing to their sequestration capacities. However, their restricted binding spectrum to 21–22-nt small RNAs and lack of discrimination between miRNAs and siRNAs limit their utility. Here we show that constitutive expression of *RNASE THREE-LIKE1* (*RTL1*) in *Arabidopsis thaliana*, previously shown to degrade siRNA precursors but not miRNA precursors, also degrades siRNA duplexes, irrespective of their size, sequence, or sub-cellular localization. Grafting experiments combined with small RNA sequencing revealed that shoot-derived siRNAs accumulate in *dcl2 dcl3 dcl4* mutant roots, but are eliminated upon ectopic *RTL1* expression, demonstrating that RTL1 efficiently degrades mobile siRNAs *in vivo*. Because the Arabidopsis endogenous *RTL1* is epigenetically silenced in wild-type plants, its ectopic expression under cell-type-specific promoters enables targeted depletion of siRNAs in selected tissues. As a proof of concept, we show that expression of *RTL1* in phloem companion cells delays systemic post-transcriptional transgene silencing (PTGS), indicating that mobile siRNAs transit through these cells. Together, these results establish RTL1 as a powerful tool to dissect siRNA mobility pathways and tissue-specific functions.

## Graphical Abstract

**Graphical Abstract:**
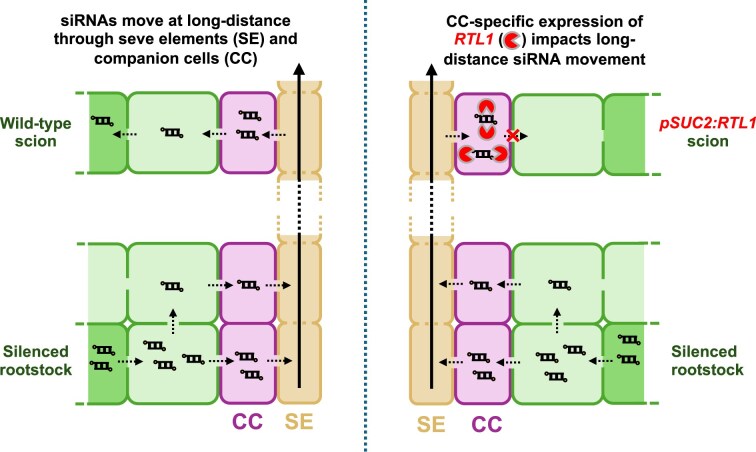

## Introduction

RNaseIII enzymes are a class of endonucleases that specifically cleave double-stranded (ds)RNA. They contain an RNaseIII signature motif composed of a highly conserved stretch of nine amino acid residues [1]. RNaseIII proteins are found in all living organisms but vary widely in length, ranging from 200 to 2000 amino acids. They are subdivided into three classes based on their domain composition [2]. Class I RNaseIII enzymes contain a single RNaseIII domain and a dsRNA-binding domain (DRB). They are mostly found in bacteria and viruses, but also in fungi, where they usually exhibit an *N*-terminal domain extension. Class II RNaseIII enzymes have one DRB and two RNaseIII domains. This class includes Drosha, which executes the first cut of miRNA precursors in animals. Finally, class III RNaseIII enzymes contain an RNA helicase domain, a PAZ domain, either one or two RNaseIII domains, and one or two DRB domains. They correspond to animal and plant Dicer, which produce small RNAs in the size range of 20–24 nt.

The plant model species *Arabidopsis thaliana* encodes four DICER-LIKE (DCL) enzymes, which are responsible for the production of micro (mi)RNAs and short interfering (si)RNAs [3, 4]. DCL1 processes PolII-derived partially self-folded single-stranded RNA molecules into 21-nt or 22-nt-long miRNAs, which are loaded mostly on the ARGONAUTE protein AGO1 to regulate complementary mRNA targets at the post-transcriptional level (PTGS). The three other DCLs produce siRNAs of different lengths from fully paired double-stranded (ds)RNA molecules whose origins are more diverse. DCL3 cleaves relatively short PolIV-derived RNAs converted into dsRNA by the RNA-dependent RNA polymerase (RDR) 2 to produce 24-nt siRNAs that are loaded on ARGONAUTE proteins of the AGO4 clade (AGO4, 6, and 9) to mediate the RNA-directed DNA methylation (RdDM) of their complementary DNA target. RdDM is mainly involved in the initiation of transposable elements (TE) transcriptional (gene) silencing (TGS). On the other hand, DCL4 and DCL2 act on longer dsRNA molecules to produce 21 nt and 22 nt siRNA, respectively, which, similar to miRNAs, are loaded into mostly AGO1 to induce PTGS on complementary RNA targets. The siRNA-dependent PTGS pathway is mainly involved in the defense against viruses and is also activated against stably integrated transgenes. For this, DCL2 and DCL4 process dsRNA molecules deriving either from viral RNA replication or from viral or transgene single-strand RNA molecules converted to dsRNA by the RNA-dependent RNA polymerase RDR6.

Besides DCL enzymes, the Arabidopsis genome also encodes several proteins that carry RNaseIII domains, either alone or associated with DRB domains. Those that carry DRB domains are referred to as RNASE THREE-LIKE (RTL) [5]. RTL1 carries one RNaseIII domain and one DRB domain. RTL2 contains one RNaseIII domain and two DRB domains, while RTL3 harbors two RNaseIII domains and three DRB domains. RTL2 is ubiquitously expressed, while RTL1 is only weakly detected in roots and in the endosperm; RTL3 is never detected in any tested tissue [5]. Arabidopsis RTL2-deficient mutants or transgenic Arabidopsis plants ectopically expressing RTL2 under the control of the constitutive *35S* promoter (*p35S*) do not show obvious developmental defects and exhibit minor changes in the small RNA repertoire [6]. In contrast, transgenic Arabidopsis plants ectopically expressing *RTL1* under the control of the constitutive *35S* promoter, accumulate miRNAs at wildtype levels but totally lack siRNAs, irrespective of their size (21-, 22-, or 24-nt), sub-cellular localization (nucleus or cytosol), and origin (endogenous or exogenous) [7]. Because siRNAs derive from perfectly paired dsRNA, whereas miRNAs derive from imperfectly paired dsRNA, these results suggest that RTL1 only targets perfectly paired dsRNA. Mutating the RNaseIII motif, deleting the DRB domain, or replacing the RTL1 DRB with an RTL2 DRB abolishes the activity of RTL1, strongly suggesting that the specificity of RTL1 for perfectly paired dsRNA is due to its DRB [6–8].

We also reported that *RTL1* expression is induced during virus infection [7, 9]. We originally thought that this induction could correspond to a defense mechanism if RTL1 was able to target dsRNA replicative forms of the viruses. However, transgenic Arabidopsis plants ectopically expressing *RTL1* under the control of the constitutive 35S promoter showed either unchanged or increased susceptibility to viruses. Cases of unchanged susceptibility correspond to viruses that express proteins referred to as viral suppressor of RNA silencing (VSR) that have the capacity to inhibit the siRNA-based PTGS defense of the plant at various steps (refs). In these cases, RTL1 could represent the only defense left to fight against viruses. However, these VSR proteins are also capable of inhibiting RTL1 activity [7]. Cases of increased susceptibility correspond to viruses that do not express VSR proteins or that express VSR proteins exhibiting only partial impairment of PTGS. In these later cases, RTL1 activity is not affected by viruses, but RTL1 fails to target dsRNA replicative forms of the viruses, likely because viruses replicate in foci that are not accessible to RTL1 [9]. In contrast, *RTL1* expression prevents the production of siRNAs directed against the viruses, thus counteracting the siRNA-based antiviral defense of the plants. These results therefore indicate that *RTL1* could be considered as a host susceptibility gene that is induced by viruses as a strategy to further limit the plant siRNA-based antiviral defense when VSRs are missing or partially active against the plant siRNA-based antiviral defense.

These results also raised questions about the reason why such a deleterious gene is maintained in the genome. To address this question, we compared wildtype and *rtl1* mutants in the only vegetative tissues where RTL1 was naturally detected, i.e. roots. We show that RTL1 is active in roots when over-expressed constitutively, but that its natural level is insufficient or restricted to a very limited number of cell types to have a visible effect on the endogenous siRNA repertoire and on siRNA-based transgene PTGS when analyzing whole root extracts. Given the absence of RTL1 activity, at least in vegetative tissues, we reasoned that RTL1 could be used as a tool to cure specific tissues from their siRNA contents by expressing *RTL1* under specific promoters. At first, we examined whether only siRNA precursors were targeted by RTL1 as proposed previously [7, 8], or if siRNAs themselves could be targeted. For this, we grafted wildtype shoots on roots expressing *RTL1* in a genetic background where siRNAs are not produced (i.e. in a *dcl2 dcl3 dcl4* triple mutant). Genome-wide sRNAseq analysis revealed that siRNAs moving from shoots to roots through the vascular tissues were found in grafted *dcl2 dcl3 dcl4* roots, but not in grafted *dcl2 dcl3 dcl4 RTL1* roots, indicating that RTL1 has the capacity to degrade mobile siRNA duplexes. Finally, we expressed *RTL1* specifically in companion cells of the phloem using the *SUCROSE PROTON-SYMPORTER* (*SUC*) *2* promoter and observed that the establishment of transgene systemic PTGS was delayed, indicating that mobile siRNA duplexes move through phloem companion cells.

## Materials and methods

### Plant material

Wild-type plants, transgenic lines, and loss-of-function mutants used in this study are in *Arabidopsis thaliana Columbia* (*Col-0*) ecotype. The *L1* and *6b4* lines carry a *p35S:GUS* transgene that is silenced or stably expressed, respectively [10, 11]. The *pSUC2:hpSUL* and *pSUC2:hpPDS* lines have been described under the name *SUC-SUL* and *JAP3* [12, 13]. The *rdr6(sgs2-1), rtl1-1* and *dcl2-1* dcl3-1 *dcl4-2* mutants have been described previously [14, 15].

### Constructs and transformation

The *p35S:RTL1* construct has been described before [7].

To generate the *pUBQ10:RTL1* construct, the *RTL1* genomic fragment described in (Shamandi *et al*., 2015) was used for an LR (Gateway Technology – Invitrogen/Thermo Fisher Scientific) reaction with the *pUB-Dest* vector [16].

To generate *pSUC2:RTL1*, the same *RTL1* genomic fragment was used for a tripartite LR reaction with *pSUC2* and the vector *pB7m24GW* (Gateway Technology – Invitrogen/Thermo Fisher Scientific).

*Agrobacterium tumefaciens* strains carrying plasmids of interest were grown overnight at 28°C in 3 ml LB medium containing the appropriate antibiotics to a final OD600 between 1 and 2. For *A.thaliana* transformation, the bacteria were pelleted and resuspended in 300 ml of infiltration medium (5% sucrose, 10mM MgCl2, 0 015% silwet L-77) to a final OD600 of 1, which was used for floral dipping.

### Growth conditions and grafting

*A.thaliana* seeds were surface-sterilized, sown on a nutritive medium (1.3% S-medium Duchefa, 1% Phytoblend agar), stratified at 4°C for 2 days, and then placed in a culture chamber at 23°C, 70% humidity, 120 µE m − 2 light with a 16 h light/8 h dark (long-days) or 8 h light/16 h dark (short-days) photoperiod. Seedlings grown under long-day conditions were transferred to soil after 2 weeks.

For grafting experiments, seedlings were grown under short-day conditions. Briefly, 6 days after germination, seedlings were cut transversely across the hypocotyl using a razor blade (90° butt graft). Then, scions and rootstocks were placed on a nitrocellulose filter (Hybond). Hypocotyls of scions and rootstock were introduced into a silicon microtube (2 mm long) to connect them to each other, and incubated under short-day conditions (8 h light, 16 h dark) for 7–14 days. Grafted seedlings that did not show adventitious roots were transferred to soil and grown under a short-day photoperiod.

### GUS assays

GUS activity was measured as described before [6]. Briefly, leaves grinded in a phosphate buffer (pH 7.2, Na2HPO_4_ 50 mM, NaH_2_PO_4_ 50 mM, EDTA 10 mM) are centrifuged for 20 min at 4°C and 3000 rpm. A Bradford protein assay was performed (Protein Assay Dye Reagent 500–0006, BioRad) with a BSA range, and protein concentration was quantified with an ELx808 microplate reader (Biotek). Enzymatic activity was measured via the derived products generated from a 4-MUG substrate (M1404, Duchefa) with a Fluoroskan Ascent II (Thermo Fisher Scientific). GUS activity is presented in an arbitrary unit as the ratio between fluorescence data per minute and protein concentration.

### RNA gel blots

RNA extraction and hybridization were performed as previously described [17]. Briefly, frozen leaves were grinded in liquid nitrogen, added to a NaCl extraction buffer (0.1M NaCl, 2% SDS, 50 mM Tris/HCl, pH 9, 10 mM EDTA, pH 8, 20 mM β-mercaptoethanol), and total RNA was extracted using a standard Phenol-Chloroform procedure. RNA was recovered in a 3v of 100% EtOH and 1/10v 3M NaOAc (pH 5.2) buffer at -80°C for 1 h. After a series of centrifugations, RNA pellets were resuspended in sterile water and quantified with a NanoDrop 2000C (Ozyme). For the low molecular weight (LMW) northern blot, 5–30 ug of RNA were denatured at 85°C for 5 min, separated on a 15% polyacrylamide, 7.5M urea, and 1X TBE gel, and transferred to a Hybond NX membrane (Amersham). For the high molecular weight (HMW) northern blot, 5 ug of RNA were denatured at 85°C for 5 min, separated on a 0.8–1.5% agarose, 0.7% formaldehyde, 20 mM HEPES, and 1 mM EDTA pH 7.8 gel, and transferred on a Genescreen Plus membrane (NEF-976, NEN/DuPont). PCR-probes were produced using primers listed in Supplementary Table S1, purified with a NucleoSpin® Gel & PCR Clean-up kit (Machery-Nagel), and radiolabeled dCTP-P^32^ was incorporated with a Prime-a-gene Labeling System kit (U1100, Promega). Oligonucleotide probes were ordered from GenoScreen, and radiolabeled dATP-P^32^ was incorporated with a T4 Polynucleotide Kinase kit (T4 PNK EK0031, Thermo Fisher Scientific). After blot saturation with salmon sperm, hybridization was performed in a PerfectHyb buffer (H7033, Sigma-Aldrich) overnight at 37°C (dCTP-P^32^ labeled probes) or at 50°C (dATP-P^32^ labeled probes) for LMW northern blot and in a Church buffer (7% SDS, 250 mM Na_2_HPO_4_, 2mM EDTA 200 μg/mL Heparin) overnight at 65°C for HMW northern blot. After exposition on a BAS-MP 2040P imaging plate (Fujifilm), the hybridization signal was revealed with a Typhoon-FLA9500 phosphoimager (Ge-Healthcare). RNA band intensity was measured on an unsaturated image with ImageJ. Data were normalized to the band intensity of the loading control.

### Small RNA-seq data collection and bioinformatic analysis

Leaves and roots of non-grafted *L1* plants, as well as roots of *dcl2 dcl3 dcl4*, and *dcl2 dcl3 dcl4 pUBQ:RTL1* non-grafted or *L1*-grafted plants were collected and used for total RNA extraction. Samples were collected from short-day-grown plants 67 days after grafting. For each condition, three different plants were sampled and processed as biological replicates. Total RNAs were sent to Singleron, which performed small RNA libraries preparation by pre-sizing the 18–30 nt RNA fraction using the BluePippin system and further sequenced the libraries.

After reception of the sequencing results, 3′ Universal Illumina adapters were trimmed and 18–32 nt reads extracted using CutAdapt (v1.14, –error-rate 0.02). Quality control of the fastq file was made before and after trimming using FastQC (v0.12.1) (http://www.bioinformatics.babraham.ac.uk/projects/fastqc/). Reads were then aligned on the *A.thaliana* reference genome TAIR10 using ShortStack (v4.0.3, –mmap u) [18]. Genome annotations used for downstream analysis correspond to the TAIR10 annotation. Samtools (v1.19) [19] was then used to extract 21 nt to 24 nt reads with a MAPQ score ≥ 10 and removed reads mapping to rRNA, tRNA, and miRNA (according to TAIR10 annotation) in order to only keep reads we assume to correspond to siRNA. Downstream analysis and plots were made with R Studio (v2025.09.0 + 387). Data parsing and processing were made with packages dplyr (v1.1.4) and data.table (v1.17.18) and plots were made with the package ggplot2 (v4.0.1). In all the plots, siRNA abundance is expressed as reads per million (RPM).

## Results

### Endogenous RTL1 expression in roots is insufficient to impact PTGS and siRNA levels

**Figure 1.**
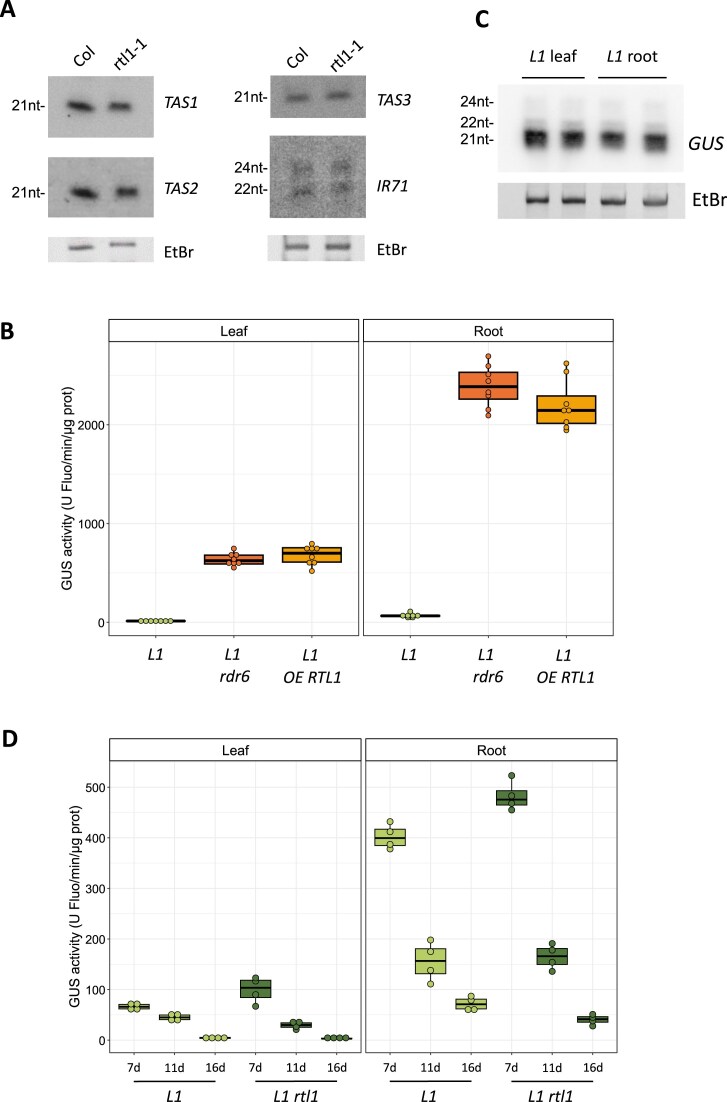
RTL1 is functional in roots but insufficiently expressed to impact siRNA-based PTGS. (**A**) RNA gel blot analysis showing the accumulation of endogenous siRNAs in 1-month-old whole roots of the indicated genotypes. Ethidium bromide staining of the gel is shown as a control. (**B**) GUS activity in leaves and whole roots of 1-month-old plants carrying the *p35S:GUS* locus *L1* in the indicated genotypes. GUS activity is in arbitrary units of fluorescence.ug of protein-1.min-1. Each dot represents an individual plant, and *n* > 7 for each genotype. (**C**) RNA gel blot analysis showing GUS siRNA accumulation in silenced plants carrying the *p35S:GUS* locus *L1*. Ethidium bromide staining of the gel is shown as a loading control. (**D**) GUS activity in leaves and whole roots of plants carrying the *p35S:GUS* locus *L1* in the indicated genotypes was harvested at 7, 11, or 16 days after germination. GUS activity is in arbitrary units of fluorescence.ug of protein-1.min-1. Each dot represents a bulk of four plants, and *n* = 4 for each genotype.

Contrasting the absence of expression of RTL1 in vegetative aerial parts, *RTL1* mRNA is detected in roots [5]. To determine if endogenous *RTL1* expression in roots could have an impact on siRNA accumulation in this tissue, the accumulation of endogenous siRNAs in roots of the *rtl1-1* null allele was compared to that of wild-type roots. Similar levels of *TAS1, TAS2, TAS3*, and *IR71* siRNAs were observed in wild-type and *rtl1-1* roots (Fig. 1A), indicating that the absence of RTL1 has no effect on siRNA accumulation when analyzing whole root extracts.

The efficiency of transgene S-PTGS was also compared between leaves and roots. Transgene S-PTGS is a process that takes place at each generation during the early development of the plant. In the Arabidopsis *p35S:GUS* line *L1*, GUS activity rapidly drops after germination to reach almost zero in adult leaves [11]. Comparing GUS activity in roots and leaves of *L1* and *L1 rdr6* plants revealed that S-PTGS causes a dramatic reduction of GUS activity in both leaves and roots (Fig. 1B). Consistently, high amounts of *GUS* siRNAs are detected in leaves and roots of *L1* plants (Fig. 1C), suggesting that *RTL1* expression in roots does not reduce S-PTGS efficiency or siRNA levels when analyzing whole root extracts. To further examine if RTL1 could have a subtle effect on S-PTGS in roots, the *L1* locus was introduced into the *rtl1-1* null allele by crossing, and the kinetics of *L1* S-PTGS were examined in *L1* and *L1 rtl1-1* shoots and roots. As expected, no difference was observed between *L1* and *L1 rtl1-1* in shoots where *RTL1* is not expressed. No difference in the kinetics of S-PTGS was also observed between *L1* and *L1 rtl1-1* in roots (Fig. 1D), indicating that RTL1 depletion has no impact on *L1* silencing when analyzing whole root extracts.

Multiple hypotheses could explain these results: (i) endogenous RTL1 expression in roots is too low to impact siRNA accumulation, (ii) endogenous RTL1 expression in roots is restricted to a very limited number of cell types (visible on https://bar.utoronto.ca/eplant/ [20] and https://bioit3.irc.ugent.be/plant-sc-atlas/root). Therefore, the impact of RTL1 depletion on endogenous siRNA accumulation or transgene S-PTGS cannot be visualized when analyzing whole root extracts, (iii) RTL1 mRNA is not translated into RTL1 protein in roots, or (iv) RTL1 protein is produced in roots but is not functional because roots lack a cofactor or specific modifications that are necessary for RTL1 activity. To discriminate between these hypotheses, a pUBQ10:RTL1 construct was introduced into the L1 line, and GUS activity was measured in shoots and roots of L1, L1 rdr6, and L1 pUBQ10:RTL1 plants (Fig. 1B). S-PTGS was suppressed in both shoots and roots of L1 pUBQ10:RTL1 plants, indicating that, in roots, RTL1 is functional, thus ruling out the third and fourth hypotheses. Therefore, the fact that RTL1 deficiency has no impact on endogenous siRNA accumulation and transgene S-PTGS efficiency in whole root extracts suggests that endogenous RTL1 expression is too low and too restricted to certain cell types to impact S-PTGS of transgenes driven by a constitutive promoter, or to modify the accumulation of endogenous siRNAs produced in a larger number of cell types (see TAS expression patterns at https://bioit3.irc.ugent.be/plant-sc-atlas/root).

### RTL1 eliminates mobile siRNAs

The results presented above, as well as previous results [7, 9] indicate that RTL1 prevents the production of siRNAs. Previous analyses revealed that RTL1 is able to cleave the dsRNA precursors of siRNAs, including the precursors of 24-nt siRNAs, whose size generally does not exceed 45-nt [7, 8]. Moreover, the accumulation of synthetic 34/36-nt dsRNAs was strongly reduced after incubation with RTL1 *in vitro* [21]. However, the possibility that RTL1 could also cleave siRNA duplexes *in vivo* has not been tested yet. Determining if RTL1 can degrade siRNA duplexes *in vivo* implies expressing *RTL1* in tissues where siRNA duplexes but not their precursors are present. This can be achieved by grafting plants expressing mobile siRNA duplexes onto plants lacking siRNAs but expressing *RTL1*.

**Figure 2.**
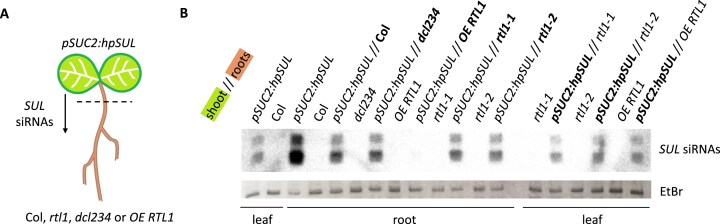
Ectopically expressed RTL1 degrades mobile siRNA duplexes of exogenous origin. (**A**) Design of the experiment. At the two cotyledon stage, scions of plants expressing the *pSUC2:hpSUL* transgene are grafted onto roots of plants of the indicated genotypes, which do not carry the *pSUC2:hpSUL* transgene, allowing mobile *SUL* siRNAs to be detected in roots. (**B**) RNA gel blot analysis showing the accumulation of *SUL* siRNAs in the *pSUC2:hpSUL*-grafted or non-grafted indicated genotypes. For each condition (grafted and non-grafted), *SUL* siRNA has been analyzed in both shoots and roots. For *pSUC2:hpSUL*-grafted plants, the part of the graft analyzed is indicated in bold. Ethidium bromide staining of the gel is shown as a control.

At first, plants expressing a transgene producing mobile siRNA duplexes were grafted onto plants expressing *RTL1*. For this purpose, shoots of the *pSUC2:hpSUL* line were grafted onto wildtype, *dcl2 dcl3 dcl4, rtl1-1, rtl1-2* or *p35S:RTL1* roots. The *pSUC2:hpSUL* line carries a transgene expressing a hairpin corresponding to a part of the *SULFUR* (*SUL*) gene under the control of the *SUC2* promoter, leading to the production of *SUL* siRNAs in the phloem companion cells [12]. Previous analyses revealed that *SUL* siRNA duplexes are highly mobile and efficiently migrate from shoots to roots [22]. Indeed, similar levels of *SUL* siRNAs were detected in wildtype and *dcl2 dcl3 dcl4* roots grafted onto *pSUC2:hpSUL* shoots (Fig. 2), indicating that *SUL* siRNA duplexes, but not the *hpSUL* dsRNA precursors of siRNAs, are moving from shoots to roots. Levels of *SUL* siRNAs were also similar in wildtype, *rtl1-1*, and *rtl1-2* roots grafted onto *pSUC2:hpSUL* shoots (Fig. 2), indicating that endogenous *RTL1* expression in roots is not sufficient to reduce the level of mobile siRNA duplexes, similar to what has been observed on siRNAs expressed in roots (Fig. 2). Contrasting the high levels of 21 nt and 24 nt *SUL* siRNAs detected in wildtype roots grafted onto *pSUC2:hpSUL* shoots, neither 21 nt nor 24 nt *SUL* siRNAs were detected in *p35S:RTL1* roots grafted onto *pSUC2:hpSUL* shoots (Fig. 2). These results not only confirm that *RTL1* functions in roots, but, more importantly, reveal that RTL1 can degrade 21 nt and 24 nt siRNA duplexes in addition to longer dsRNA precursors.

**Figure 3.**
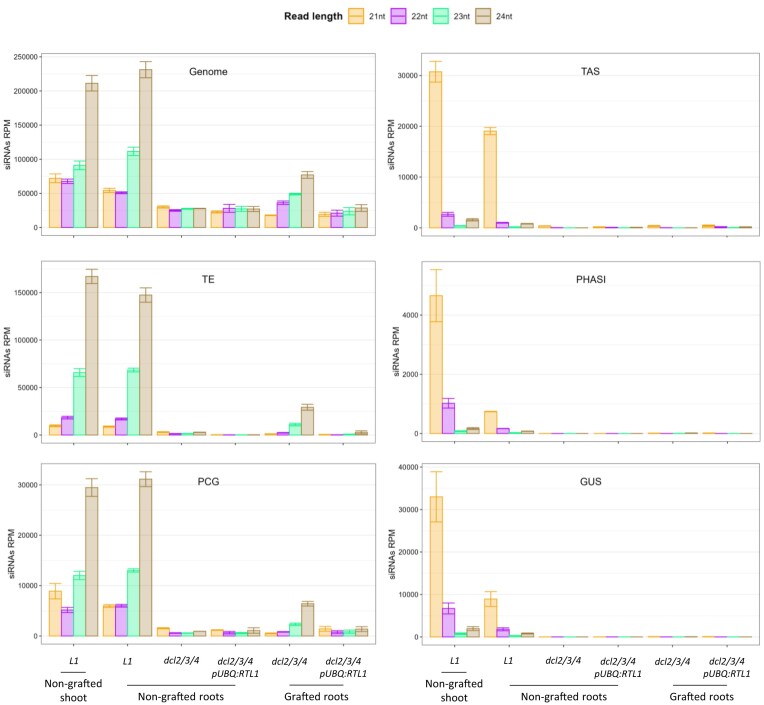
Ectopically expressed RTL1 degrades mobile siRNAs of endogenous origin. Small RNAs were extracted from non-grafted leaves or roots of wildtype *(p35S:GUS L1)* plants, *dcl2 dcl3 dcl4 (dcl2/3/4)* triple mutant or *dcl2 dcl3 dcl4* plants expressing a *pUBQ:RTL1* transgene *(dcl2/3/4 OE RTL1,)* or roots of *dcl2/3/4* or *dcl2/3/4 OE RTL1* plants grafted on wildtype *(p35S:GUS L1)* shoots, and subjected to high throughput sequencing. Experiments were performed in triplicate. siRNA size distribution is shown for the whole genome (Genome), protein-coding genes (PCG), transposable elements (TE), non-coding genes producing phased siRNAs upon clivage by a miRNA (TAS), coding genes producing phased siRNAs upon clivage by a miRNA (PHASI), and the bacterial sequence encoded by the *p35S:GUS* transgene (GUS). siRNAs correspond to reads that perfectly match the *A.thaliana* nuclear genome. excluding miRNA, rRNA, and tRNA. The proportion of each size of siRNA is indicated by a different color. Values are expressed in reads per million (rpm).

To determine if RTL1 can degrade any type of siRNA duplexes irrespective of their sequence, the impact of RTL1 on mobile siRNAs was examined genome-wide. For this, we grafted shoots of the *p35S:GUS* line *L1* (in a WT background) on roots of the *pUBQ10:RTL1* line in a genetic background where siRNAs are not produced (i.e. in a *dcl2 dcl3 dcl4* triple mutant). Genome-wide sRNAseq analysis confirmed that a significant proportion of endogenous siRNAs move from wildtype (*L1)* shoots to *dcl2 dcl3 dcl4* roots through the graft union. In line with previous studies [23, 24], the majority of endogenous siRNAs moving from scion to rootstock corresponds to 23 nt and 24 nt siRNAs (Fig. 3). A close-up on siRNA origins showed that 23 nt/24 nt siRNAs moving from wildtype scions to *dcl2 dcl3 dcl4* rootstocks come from transposable elements (TEs) and protein-coding genes (PCGs) (Fig. 3). In contrast, very few 21 nt/22 nt siRNAs move from wildtype scions to *dcl2 dcl3 dcl4* rootstocks (Fig. 3). Indeed, analysis of *L1* siRNAs and tasiRNAs, which represent the main producers of 21 nt/22 nt siRNA in shoots, showed no specific enrichment of 21 nt/22 nt siRNA in recipient *dcl2 dcl3 dcl4* rootstocks, while they together account for more than 55% of this type of siRNA in wildtype (*L1)* shoots (Fig. 3). The profile of phased secondary 21 nt/22 nt siRNAs referred to as tasiRNAs and phasiRNAs, depending on whether they derive from the cleavage of coding (phasi) or non-coding (tasi) mRNA by a 22 nt miRNA [3, 4], followed the same behavior (Fig. 3). Finding that 23 nt/24 nt siRNAs move more efficiently than 21 nt/22 nt siRNAs through a graft-union was consistent with previous reports [23, 24]. This over-representation of 23 nt/24 nt mobile siRNAs compared to 21 nt/22 nt siRNAs in grafted *dcl2 dcl3 dcl4* rootstocks suggests that 21 nt/22 nt siRNA species specifically loaded into PTGS-related AGOs (i.e. mostly AGO1 and AGO2) are mainly consumed where their RNA targets are produced, allowing only a slight and almost undetectable amount to move through vascular tissues, a process previously referred to as consumption [22]. In contrast to *dcl2 dcl3 dcl4* rootstocks grafted onto wildtype (*L1)* shoots, which accumulate mobile siRNAs, siRNAs were found at a background level in grafted *dcl2 dcl3 dcl4 pUBQ:RTL1* roots (Fig. 3), indicating that RTL1 has the capacity to degrade all mobile siRNA duplexes. Indeed, all classes of siRNAs derived from TEs, PCGs, *TAS, PHAS*, and *GUS* loci were absent, indicating that RTL1 degrades mobile siRNAs, irrespective of their size or origins.

### Ectopic RTL1 expression can eliminate siRNAs in a tissue-specific manner

**Figure 4.**
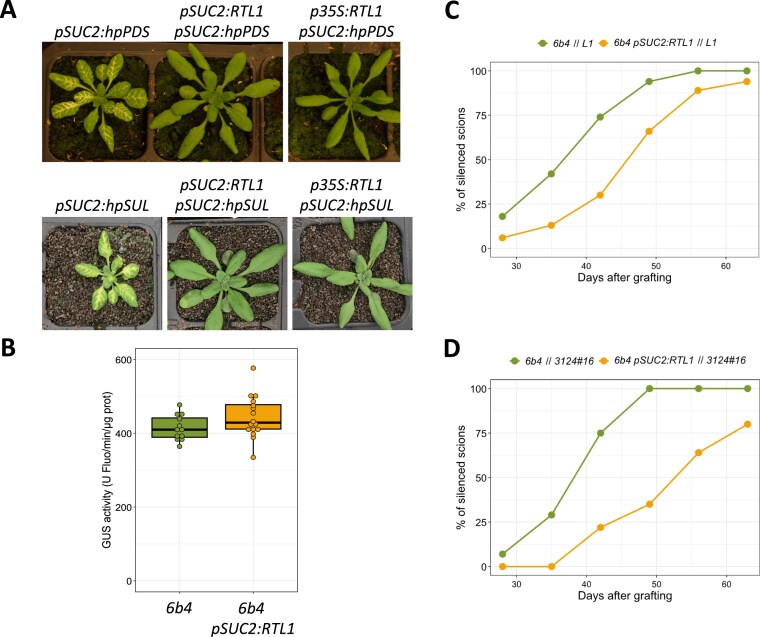
RTL1 expression in phloem companion cells delays S-PTGS systemic propagation. (**A**) Pictures of plants expressing *p35S:RTL1* or *pSUC2:RTL1* in *pSUC2:hpPDS* or *pSUC2:hpSUL* lines. (**B**) GUS activity in leaves of 1-month-old plants of the non-silenced *p35S:GUS* line *6b4*, carrying or not the *pSUC2:RTL1* locus derived from the *pSUC2:hpPDS/pSUC2:RTL1* line showing the most efficient suppression of the *pSUC2:hpPDS* phenotype (shown in A). GUS activity is in arbitrary units of fluorescence.ug of protein^−1^.min^−1^. (**C**) Time course of S-PTGS establishment in non-silenced scions of lines *6b4* grafted onto silenced *p35S:GUS* (*L1)* rootstocks (*6b4 // L1, n* = 50) or *6b4 pSUC2:RTL1* scions grafted onto L1 rootstocks (*6b4 pSUC2:RTL1 // L1, n* = 47). Grafted scions are considered silenced when *GUS* expression is less than 5% of non-grafted controls. (**D**) Time course of S-PTGS establishment in non-silenced scions of lines *6b4* grafted onto silenced *pUBQ10:GFP-GUS (3124#16)* rootstocks (*6b4 // 3124#16, n* = 28) or *6b4 pSUC2:RTL1* scions grafted onto *3124#16* rootstocks (*6b4 pSUC2:RTL1 // 3124#16, n* = 27). Grafted shoots are considered silenced when GUS expression is less than 5% of non-grafted controls.

The results presented above indicate that constitutive expression of *RTL1* under the control of the *35S* or *UBQ10* promoter prevents the accumulation of endogenous or transgenic siRNAs in every tissue. To examine if cell-specific *RTL1* expression could be used to eliminate siRNAs from specific cell types, a *pSUC2:RTL1* transgene was generated and introduced into the *pSUC2:hpSUL* and *pSUC2:hpPDS* lines. Similar to the *pSUC2:hpSUL* line that carries a transgene producing *SUL* siRNAs in the phloem companion cells, which induces *SUL* silencing in the layer of cells surrounding the phloem [12], the *pSUC2:hpPDS* line carries a transgene that produces *PDS* siRNAs in the phloem companion cells, resulting in *PDS* silencing in the layer of cells surrounding the phloem [13]. Expressing *RTL1* specifically in the phloem companion cells using the *pSUC2:RTL1* transgene suppressed *PDS* and *SUL* PTGS as efficiently as expressing a *p35S:RTL1* transgene (Fig. 4A), indicating that cell-specific expression of RTL1 at the site of dsRNA production is sufficient to destroy dsRNA and siRNA duplexes before they move to adjacent cells.

### RTL1 expression in phloem companion cells delays systemic S-PTGS propagation

During transgene S-PTGS, silencing is initiated locally and subsequently becomes systemic, due to siRNA propagation within the plant. Transgene siRNA duplexes can move from cell to cell through plasmodesmata, but also at long distances. This last movement was demonstrated by grafting scions of lines that never trigger S-PTGS spontaneously onto rootstocks of lines that spontaneously undergo S-PTGS, allowing S-PTGS to be established in the grafted scions [26–28]. This long-distance propagation cannot be due only to the movement of siRNA duplexes from cell to cell. Indeed, three-part grafting experiments using a piece of wild-type tobacco stem interspersed between a silenced rootstock and a non-silenced target scion revealed long-distance movement through the phloem [27]. For this to happen, siRNA duplexes need to be loaded from source cells into the phloem and subsequently unloaded in sink cells. Because phloem companion cells (PCCs) were proposed to play a key role in siRNA movement and in the systemic aspect of S-PTGS [25], grafting experiments were carried out using an Arabidopsis line expressing *RTL1* in the phloem companion cells in order to destroy mobile siRNA duplexes specifically in this cell type. For this purpose, the *pSUC2:hpPDS pSUC2:RTL1* transgenic line that suppresses *PDS* PTGS the most efficiently was crossed to the *p35S:GUS* line *6b4*, which never triggers S-PTGS spontaneously, but has the capacity to undergo S-PTGS upon grafting onto rootstocks of the *p35S:GUS* line *L1*, which spontaneously undergoes S-PTGS [28]. F2 plants homozygous for the *pSUC2:RTL1* locus and the 6b4 locus, and that have eliminated the *pSUC2:hpPDS* locus, were selected. At first, GUS activity was compared in the different lines. As expected, no difference was observed between *6b4* and *6b4 pSUC2:RTL1* plants (Fig. 4B). Then, the efficiency of graft-induced S-PTGS propagation was examined in *6b4* and *6b4 pSUC2:RTL1* scions grafted onto *L1* rootstocks. An important delay in the propagation of S-PTGS was observed in *6b4 pSUC2:RTL1* shoots grafted onto *L1* roots (Fig. 4C), indicating a contribution of phloem companion cells in systemic S-PTGS. Nevertheless, all grafted scions eventually triggered systemic S-PTGS, suggesting either that RTL1 is not sufficiently expressed under the pSUC2 promoter to totally eliminate mobile siRNA duplexes from all companion cells or that mobile siRNA duplexes can be unloaded from the phloem through other cell types.

To further examine the role of phloem companion cells in systemic S-PTGS, *6b4 pSUC2:RTL1* shoots were grafted onto the roots of a second transgenic line undergoing *GUS* S-PTGS but based on a different transgenic system. For this purpose, *6b4* and *6b4 pSUC2:RTL1* shoots were grafted onto the roots of line *pUBQ10:GFP-GUS#20*. As previously observed for *6b4 pSUC2:RTL1* shoots grafted onto *L1* roots, the establishment of S-PTGS in *6b4 pSUC2:RTL1* shoots grafted onto pUBQ10:GFP-GUS#20 roots was delayed compared to that of grafted 6b4 shoots, and a fraction of the plants did not trigger S-PTGS at all (Fig. 4D), confirming that the ablation of *GUS* siRNAs in phloem companion cells strongly impacts the systemic aspect of S-PTGS.

## Discussion

RTL1 is the simplest RNaseIII enzyme encoded by the Arabidopsis genome [5]. It comprises a unique RNaseIII domain and a unique DRB domain, which likely specializes this enzyme to recognize and cleave only perfectly paired dsRNA. Indeed, *RTL1* over-expression prevents the accumulation of all siRNAs, but not of miRNAs, which derive from imperfectly paired dsRNA [7]. Because it prevents the accumulation of every siRNA produced from the 13 000 siRNA-producing loci [7], RTL1 likely acts in a structure-dependent but sequence-independent manner. The analysis of plants that express *RTL1* in a *dcl2 dcl3 dcl4* triple mutant, in which siRNA precursors accumulate because they cannot be processed, previously revealed that RTL1 cleaves long dsRNA [7]. We and others also showed that RTL1 is able to cleave shorter dsRNA *in vitro* [7, 8, 21]. However, it has long remained unclear if RTL1 was able to cleave siRNA duplexes *in vivo* in addition to cleaving their precursors. Indeed, addressing this question implies expressing *RTL1* in cells accumulating siRNAs but where their precursors are not produced. Separating the site of dsRNA production from the site of siRNA accumulation can be achieved by grafting wild-type plants onto *dcl2 dcl3 dcl4* triple mutants because siRNA duplexes, but not their precursors, have the capacity to move through vascular tissues [22–24]. Therefore, grafting wildtype plants onto *dcl2 dcl3 dcl4* triple mutants expressing *RTL1* allows determining if RTL1 can target siRNA duplexes unambiguously. The genome-wide analysis performed in this study reveals that RTL1 is capable of destroying every siRNA duplex (Figs 2 and 3), which confirms its sequence-independent activity. RTL1’s ability to destroy siRNA duplexes in addition to their precursors indicates that RTL1 does not need to compete with DCL2, DCL3, and DCL4 to bind to siRNA precursors and destroy them before they are processed by the DCLs. Indeed, even if a fraction of siRNA precursors escape destruction by RTL1 and remain processed by DCL2, DCL3, or DCL4, the resulting siRNAs are subsequently destroyed by RTL1, which explains why plants over-expressing *RTL1* phenocopy *dcl2 dcl3 dcl4* triple mutants and lack all siRNAs [7].

Despite RTL1’s capacity to destroy siRNA duplexes or precursors, wildtype Arabidopsis plants accumulate mature siRNAs, suggesting that wildtype plants lack RTL1 activity (Fig. 1). Indeed, RTL1 is naturally not expressed in vegetative tissues except in roots. However, the results obtained by comparing wildtype plants, *rtl1* mutants, and plants overexpressing *RTL1* indicate that RTL1 is functional in roots but that its native level of expression is insufficient to affect the siRNA repertoire or the siRNA-dependent PTGS capacity of the plant. Therefore, one could consider that *RTL1* is not expressed at all in vegetative tissues under normal conditions, and that it plays no role in the siRNA homeostasis. Only when *RTL1* is induced in vegetative tissues during virus infection does it appear to play a detectable role. However, this role appears mostly deleterious to the plant because destroying the siRNAs produced by the antiviral PTGS defense of the plant promotes viral infection [7, 9]. Therefore, the reason why this gene is maintained in the Arabidopsis genome remains unknown. Whether it has a positive or adaptive role remains to be determined. *RTL1* expression is also detected in the endosperm, an essential tissue of the seed where genomic expression is highly reprogrammed. Therefore, it is possible that *RTL1* expression is required in this tissue. However, *rtl1* mutants are perfectly fertile, leaving open this question.

The fact that Arabidopsis vegetative tissues do not express *RTL1* or express it at levels insufficient to degrade siRNA duplexes makes it a fantastic tool to investigate the role of siRNAs in each cell type and at each stage of development by expressing it under specific promoters, thus allowing to specifically eliminate siRNAs at a given time and in a given cell type. So far, small RNA neutralization could only be achieved using virus-encoded proteins that sequestrate small RNAs such as P19, Hc-Pro, P21, p15, p130/p126/p122, γB, NS3, Pns10, and NSs (reviewed in [29]). The most characterized is the tombusviral P19 protein, which acts as a homodimer to size-select and sequester small RNA duplexes in a sequence-independent manner [30, 31]. As a result, P19 binds only to 21–22-nt small RNAs, including miRNAs and siRNAs, but not to longer ones. In particular, it does not bind to 24-nt siRNAs, which represent the largest class of siRNAs in plants. Moreover, because P19 sequestrates both miRNAs and siRNAs, its expression results in pleiotropic defects owing to the widespread role of miRNAs. Consequently, P19 is mostly used in transient assays to prevent the silencing of the extra-chromosomal copies of the introduced transgene, and not for making stable transformants [32]. Ectopic *RTL1* expression therefore provides a unique tool for specifically eliminating siRNAs, irrespective of their size, and without unintended side-effects on the miRNA pathway. Previous work showed that expressing *RTL1* under the control of promoters specific to the sperm cell and vegetative nucleus eliminates distinct populations of siRNAs in pollen [33]. However, it was not clear whether siRNA precursors or mature siRNAs were destroyed, and if mobile siRNAs were still exchanged between the different compartments. The work we described here reveals that both siRNA precursors and siRNA duplexes are targeted by RTL1, allowing us to use RTL1 for unambiguous cell-specific ablation of siRNAs. P19 siRNA sequestration was shown to work *in vitro* in the Drosophila embryo extract system [34]. Whether RTL1 could also function in heterologous systems remains to be determined.

One of the key questions concerning PTGS in plants is how siRNA duplexes move over long distances. It is clear that siRNAs circulate in the phloem, but the exact cell-type(s) allowing siRNA loading in and unloading from the phloem are unknown. Phloem companion cells (PCCs) were proposed to be instrumental in this process [25] To determine if PCCs actually are involved, we expressed *RTL1* specifically in this cell type. At first, we verified that RTL1 is active in this cell type by expressing *RTL1* in the PCCs of plants producing mobile siRNAs specifically in this tissue. The action at a distance of such siRNAs was blocked by RTL1, indicating that RTL1 is active in phloem companion cells and sufficiently expressed to destroy siRNA duplexes before they can move to adjacent cells (Fig. 4A). Then, *RTL1* was expressed in phloem companion cells of a plant expressing a *p35S:GUS* reporter that naturally is not silenced but can undergo PTGS upon grafting on a silenced rootstock providing PTGS-initiating siRNAs through the phloem. PTGS transmission from a rootstock exhibiting robust (*p35S*-driven) silencing to an *RTL1*-expressing non-silenced scion was delayed (Fig. 4C), indicating that phloem companion cells actually play an important role in the long-distance movement of siRNA duplexes. The late establishment of systemic S-PTGS may be due to the fact that pSUC2 expression is low in sink leaves compared to source leaves [35–37]. Accordingly, RTL1 may be sufficiently expressed in source leaves to degrade all mobile siRNA duplexes, but not in sink leaves, allowing limited amounts of mobile siRNA duplexes to enter this part of the scion and eventually trigger PTGS in mesophyll cells, although in a delayed manner. Alternatively, but not exclusively, one cannot totally rule out that mobile siRNA duplexes use alternative routes and are loaded in and unloaded from the phloem through other cell types. Nevertheless, grafting experiments performed with a rootstock exhibiting a weaker (*pUBQ10*-driven) silencing showed further delay in the transmission of PTGS to non-silenced scions, with some scions actually remaining non-silenced (Fig. 4D). This last result supports the hypothesis that siRNA duplexes move exclusively through phloem companion cells, but that a *pSUC2:RTL1* construct is insufficiently expressed in sink leaves to degrade all mobile siRNA duplexes, especially if they are produced in very large amounts, for example using a p*35S*-driven transgene as a source of mobile siRNA duplexes.

## Supplementary Material

gkag388_Supplemental_File

## Data Availability

sRNA-seq data are available at GEO under the accession number GSE318321

## References

[1] Blaszczyk J, Tropea JE, Bubunenko M et al. Crystallographic and modeling studies of RNase III suggest a mechanism for double-stranded RNA cleavage. Structure. 2001;9:1225–36. 10.1016/S0969-2126(01)00685-2 11738048

[2] MacRae IJ, Doudna JA. Ribonuclease revisited: structural insights into ribonuclease III family enzymes. Curr Opin Struct Biol. 2007;17:138–45. 10.1016/j.sbi.2006.12.002 17194582

[3] Vaucheret H, Voinnet O. The plant siRNA landscape. Plant Cell. 2024;36:246–75. 10.1093/plcell/koad253 37772967 PMC10827316

[4] Zhan J, Meyers BC. Plant small RNAs: their biogenesis, regulatory roles, and functions. Annu. Rev Plant Biol. 2023;74:21–51. 10.1146/annurev-arplant-070122-035226 36854480

[5] Comella P, Pontvianne F, Lahmy S et al. Characterization of a ribonuclease III-like protein required for cleavage of the pre-rRNA in the 3'ETS in Arabidopsis. Nucleic Acids Res. 2008;36:1163–75. 10.1093/nar/gkm1130 18158302 PMC2275086

[6] Elvira-Matelot E, Hachet M, Shamandi N et al. Arabidopsis RNASE THREE LIKE2 modulates the expression of protein-coding genes via 24-nucleotide small interfering RNA-directed DNA methylation. Plant Cell. 2016;28:406–25. 10.1105/tpc.15.00540 26764378 PMC4790866

[7] Shamandi N, Zytnicki M, Charbonnel C et al. Plants encode a general siRNA suppressor that is induced and suppressed by viruses. PLoS Biol. 2015;13:e1002326. 10.1371/journal.pbio.1002326 26696443 PMC4687873

[8] Charbonnel C, Niazi AK, Elvira-Matelot E et al. The siRNA suppressor RTL1 is redox-regulated through glutathionylation of a conserved cysteine in the double-stranded-RNA-binding domain. Nucleic Acids Res. 2017;45:11891–907. 10.1093/nar/gkx820 28981840 PMC5714217

[9] Sehki H, Yu A, Elmayan T et al. TYMV and TRV infect *Arabidopsis thaliana* by expressing weak suppressors of RNA silencing and inducing host RNASE THREE LIKE1. PLoS Pathog. 2023;19:e1010482. 10.1371/journal.ppat.1010482 36696453 PMC9901757

[10] Beclin C, Boutet S, Waterhouse P et al. A branched pathway for transgene-induced RNA silencing in plants. Curr Biol. 2002;12:684–8. 10.1016/S0960-9822(02)00792-3 11967158

[11] Elmayan T, Balzergue S, Beon F et al. Arabidopsis mutants impaired in cosuppression. Plant Cell. 1998;10:1747–57. 10.1105/tpc.10.10.1747 9761800 PMC143939

[12] Himber C, Dunoyer P, Moissiard G et al. Transitivity-dependent and -independent cell-to-cell movement of RNA silencing. EMBO J. 2003;22:4523–33. 10.1093/emboj/cdg431 12941703 PMC202373

[13] Smith LM, Pontes O, Searle I et al. An SNF2 protein associated with nuclear RNA silencing and the spread of a silencing signal between cells in Arabidopsis. Plant Cell. 2007;19:1507–21., 10.1105/tpc.107.051540 17526749 PMC1913737

[14] Henderson IR, Zhang X, Lu C et al. Dissecting *Arabidopsis thaliana* DICER function in small RNA processing, gene silencing and DNA methylation patterning. Nat Genet. 2006;38:721–5. 10.1038/ng1804 16699516

[15] Mourrain P, Beclin C, Elmayan T et al. Arabidopsis SGS2 and SGS3 genes are required for posttranscriptional gene silencing and natural virus resistance. Cell. 2000;101:533–42. 10.1016/S0092-8674(00)80863-6 10850495

[16] Grefen C, Donald N, Hashimoto K et al. A ubiquitin-10 promoter-based vector set for fluorescent protein tagging facilitates temporal stability and native protein distribution in transient and stable expression studies. Plant J. 2010;64:355–65. 10.1111/j.1365-313X.2010.04322.x 20735773

[17] Mallory AC, Hinze A, Tucker MR et al. Redundant and specific roles of the ARGONAUTE proteins AGO1 and ZLL in development and small RNA-directed gene silencing. PLoS Genet. 2009;5:e1000646. 10.1371/journal.pgen.1000646 19763164 PMC2730571

[18] Axtell MJ . ShortStack: comprehensive annotation and quantification of small RNA genes. RNA. 2013;19:740–51. 10.1261/rna.035279.112 23610128 PMC3683909

[19] Danecek P, Bonfield JK, Liddle J et al. Twelve years of SAMtools and BCFtools. Gigascience. 2021;10:giab008. 10.1093/gigascience/giab008 33590861 PMC7931819

[20] Ryu KH, Huang L, Kang HM et al. Single-cell RNA sequencing resolves molecular relationships among individual plant cells. Plant Physiol. 2019;179:1444–56. 10.1104/pp.18.01482 30718350 PMC6446759

[21] Tschopp MA, Iki T, Brosnan CA et al. A complex of Arabidopsis DRB proteins can impair dsRNA processing. RNA. 2017;23:782–97. 10.1261/rna.059519.116 28232389 PMC5393186

[22] Devers EA, Brosnan CA, Sarazin A et al. Movement and differential consumption of short interfering RNA duplexes underlie mobile RNA interference. Nat Plants. 2020;6:789–99. 10.1038/s41477-020-0687-2 32632272

[23] Lewsey MG, Hardcastle TJ, Melnyk CW et al. Mobile small RNAs regulate genome-wide DNA methylation. Proc Natl Acad Sci USA. 2016;113:E801–810. 10.1073/pnas.1515072113 26787884 PMC4760824

[24] Molnar A, Melnyk CW, Bassett A et al. Small silencing RNAs in plants are mobile and direct epigenetic modification in recipient cells. Science. 2010;328:872–5. 10.1126/science.1187959 20413459

[25] Voinnet O . Three decades of mobile RNA silencing within plants: what have we learnt?. J Exp Bot. 2026;; 77:799–817. 10.1093/jxb/eraf312. 40629501 PMC12835823

[26] Brosnan CA, Mitter N, Christie M et al. Nuclear gene silencing directs reception of long-distance mRNA silencing in Arabidopsis. Proc Natl Acad Sci USA. 2007;104:14741–6. 10.1073/pnas.0706701104 17785412 PMC1964546

[27] Palauqui JC, Elmayan T, Pollien JM et al. Systemic acquired silencing: transgene-specific post-transcriptional silencing is transmitted by grafting from silenced stocks to non-silenced scions. EMBO J. 1997;16:4738–45. 10.1093/emboj/16.15.4738 9303318 PMC1170100

[28] Taochy C, Gursanscky NR, Cao J et al. A genetic screen for impaired systemic RNAi highlights the crucial role of DICER-LIKE 2. Plant Physiol. 2017;175:1424–37. 10.1104/pp.17.01181 28928141 PMC5664484

[29] Csorba T, Kontra L, Burgyan J. viral silencing suppressors: tools forged to fine-tune host-pathogen coexistence. Virology. 2015;479-480:85–103. 10.1016/j.virol.2015.02.028 25766638

[30] Silhavy D, Molnar A, Lucioli A et al. A viral protein suppresses RNA silencing and binds silencing-generated, 21- to 25-nucleotide double-stranded RNAs. EMBO J. 2002;21:3070–80. 10.1093/emboj/cdf312 12065420 PMC125389

[31] Vargason JM, Szittya G, Burgyan J et al. Size selective recognition of siRNA by an RNA silencing suppressor. Cell. 2003;115:799–811. 10.1016/S0092-8674(03)00984-X 14697199

[32] Jay F, Brioudes F, Voinnet O. A contemporary reassessment of the enhanced transient expression system based on the tombusviral silencing suppressor protein P19. Plant J. 2023;113:186–204. 10.1111/tpj.16032 36403224 PMC10107623

[33] Pachamuthu K, Simon M, Borges F. Targeted suppression of siRNA biogenesis in Arabidopsis pollen promotes triploid seed viability. Nat Commun. 2024;15:4612. 10.1038/s41467-024-48950-6 38816386 PMC11139921

[34] Lakatos L, Csorba T, Pantaleo V et al. Small RNA binding is a common strategy to suppress RNA silencing by several viral suppressors. EMBO J. 2006;25:2768–80. 10.1038/sj.emboj.7601164 16724105 PMC1500863

[35] Imlau A, Truernit E, Sauer N. Cell-to-cell and long-distance trafficking of the green fluorescent protein in the phloem and symplastic unloading of the protein into sink tissues. Plant Cell. 1999;11:309–22. 10.1105/tpc.11.3.309 10072393 PMC144181

[36] Stadler R, Lauterbach C, Sauer N. Cell-to-cell movement of green fluorescent protein reveals post-phloem transport in the outer integument and identifies symplastic domains in Arabidopsis seeds and embryos. Plant Physiol. 2005;139:701–12. 10.1104/pp.105.065607 16169962 PMC1255989

[37] Truernit E, Sauer N. The promoter of the Arabidopsis thaliana SUC2 sucrose-H+ symporter gene directs expression of beta-glucuronidase to the phloem: evidence for phloem loading and unloading by SUC2. Planta. 1995;196:564–70. 10.1007/BF00203657 7647685

